# Conspicuity of breast lesions at different b values on diffusion-weighted imaging

**DOI:** 10.1186/1471-2407-12-334

**Published:** 2012-08-02

**Authors:** Xin Chen, Xi-Jing He, Rui Jin, You-Min Guo, Xian Zhao, Hua-Feng Kang, Li-Ping Mo, Qian Wu

**Affiliations:** 1 Department of Radiology, Second Affiliated Hospital of Medical College of Xi'an Jiaotong University, Xiwu Road, Xi'an, Shannxi, China; 2 Department of Orthopedic Surgery, Second Affiliated Hospital of Medical College of Xi'an Jiaotong University, Xiwu Road, Xi'an, Shannxi, China; 3 Department of Oncology, Second Affiliated Hospital of Medical College of Xi'an Jiaotong University, Xiwu Road, Xi'an, Shannxi, China; 4 Department of Pathology, Medical College of Xi'an Jiaotong University, Yanta Road, Xi'an, Shannxi, China; 5 Department of Epidemiology, Medical College of Xi'an Jiaotong University, Yanta Road, Xi'an, Shannxi, China

## Abstract

**Background:**

Diffusion-weighted (DW) imaging has shown potential to differentiate between malignant and benign breast lesions. However, different b values have been used with varied sensitivity and specificity. This study aims to prospectively evaluate the influence of b value on the detection and assessment of breast lesions.

**Methods:**

Institutional review board approval and informed patient consent were obtained. Between February 2010 and September 2010, sixty women suspected of having breast cancer by clinical examination and mammography underwent bilateral breast MRI and DW imaging (with maximum b values of 600, 800, and 1000 s/mm^2^). Conspicuity grades of lesions at different b values on DW images were performed. Signal intensity and apparent diffusion coefficient (ADC) values were recorded and compared among different b values by the signal-to-noise ratio (SNR), contrast-to-noise ratio (CNR) and receiver operating characteristic (ROC) curve.

**Results:**

Fifty-seven lesions from 52 recruited patients including 39/57 (68%) malignant and 18/57 (32%) benign were confirmed with pathology. DCE MRI accurately detected 53 lesions with the sensitivity of 93.0% and specificity of 66.7%, and DW imaging accurately detected 51 lesions with the sensitivity of 89.5% and specificity of 100%. There were no significant differences in conspicuity grades compared among the three b values (*P* = 0.072), although the SNR and CNR of breast lesions decreased significantly with higher b values. Mean ADCs of malignant lesions (b = 600 s/mm^2^, 1.07 ± 0.26 × 10^-3^ mm^2^/s; b = 800 s/mm^2^, 0.96 ± 0.22 × 10^-3^ mm^2^/s; b = 1000 s/mm^2^, 0.92 ± 0.26 × 10^-3^ mm^2^/s) were significantly lower than those of benign lesions (b = 600 s/mm^2^, 1.55 ± 0.40 × 10^-3^ mm^2^/s; b = 800 s/mm^2^, 1.43 ± 0.38 × 10^-3^ mm^2^/s; b = 1000 s/mm^2^, 1.49 ± 0.38 × 10^-3^ mm^2^/s) with all *P* values <0.001, but there were no significant differences among the three b values (*P* = 0.303 and 0.840 for malignant and benign lesions, respectively). According to the area under the ROC curves, which were derived from ADC and differentiate malignant from benign lesions, no significant differences were found among the three b values (*P* = 0.743).

**Conclusions:**

DW imaging is a potential adjunct to conventional MRI in the differentiation between malignant and benign breast lesions. Varying the maximum b value from 600 to 1000 s/mm^2^ does not influence the conspicuity of breast lesions on DW imaging at 1.5 T.

## Background

Magnetic resonance imaging (MRI) is becoming a powerful tool to help with the detection, diagnosis, and staging of breast cancer [1-3]. Based on the morphology and enhancement pattern of lesions, contrast-enhanced MRI offers an overall sensitivity of 90% and specificity of 72% in detecting breast lesions according to a published meta-analysis [2]. Therefore the classification of a breast lesion as malignant or benign still has its shortcomings when detected with MRI [2,4].

In recent years, diffusion-weighted (DW) imaging has been applied to distinguish between malignant and benign breast lesions [5-11]. DW imaging allows for noninvasive characterization of biologic tissues based on their water diffusion properties. Additionally, it provides information about the biophysical properties of tissues, such as cell organization and density, microstructure, and microcirculation. DW imaging enables the quantitative evaluation of apparent diffusion coefficient (ADC), an important parameter since a lower ADC has been associated with malignancy (7‐11). This occurs because of restricted water movement in high cellularity tumours (5,12). ADC may be an effective parameter in distinguishing between malignant and benign breast lesions. However, sensitivity and specificity values vary and range from 62.5% to 92.8% and 45.8% to 96.7%, respectively [13]. The reported mean ADC of malignant lesions ranges from 0.90 to 1.61 × 10^-3^ mm^2^/s and that of benign lesions from 1.41 to 2.01 × 10^-3^ mm^2^/s. These distributions have resulted in a recommended ADC cutoff value ranging from 1.1 to 1.6 × 10^-3^ mm^2^/s between malignant and benign lesions [14]. Comparison of the diagnostic performance of breast DW imaging among the studies has been compromised by the lack of standardization [15-17].

On clinical MRI scanners, diffusion sensitivity is easily altered by changing the diffusion gradient factor, which is also known as the “b value”. This value is proportional to the gradient amplitude and duration. By using at least two different b values, the ADC can be calculated. Tsushima *et al.*[14] reported that the maximum b value (b_max_) correlated with the mean ADC of malignant and benign tumours. In previous studies, it should be noted that b_max_ ranged from 300 to 1000 s/mm^2^, with a total of two to five values in between [18]. For DW imaging, a higher b value provides more tissue diffusivity and less T_2_ shine-through effect [19,20]. However, higher b values may result in poor image quality due to an inferior signal-to-noise ratio (SNR) at 1.5 T [21,22]. Therefore, Matsuoka *et al.* proposed a systematic evaluation of b values [23]. The optimum b value should sufficiently suppress the background signal of the glandular parenchyma and provide a lesion signal that is strong enough to allow image interpretation. Additionally, it should differentiate malignant and benign lesions with the best accuracy.

Based on these concepts we have carried out a prospective study to evaluate the influence of different b values on lesion conspicuity, the ADC measurement and the performance of ADC for the differential diagnosis between malignant and benign breast lesions.

## Methods

### Patients

The study was approved by our institutional review board. Informed consent was obtained from all patients before participation in the study. Sixty patients with palpable breast mass and clinical indication for breast MRI were enrolled from February 2010 to September 2010. Three patients were excluded because of movement or susceptibility artifacts seen on DW imaging. Two additional patients were lost without sufficient follow-up. Thus, 55 women (mean age, 49.6 years; range, 23-75 years) who met the following inclusion criteria were included in our study. 1) Insufficient or suspicious mammography findings (breast mass, architectural distortion, and focal asymmetry) diagnosed as Breast Imaging Reporting and Data System (BI-RADS) 0, 4, 5; 2) The final diagnosis was confirmed by means of surgery or core needle biopsy; 3) Clinical and imaging follow-ups were performed for at least 12 months by using a combination of ultrasound and mammography.

### MRI protocol

MRI examination was performed with a 1.5 T system (Signa; GE Medical Systems, Milwaukee, Wisconsin) using a dedicated four-channel breast coil on patients in the prone position. The imaging protocol began with standard anatomical series, including fast spin-echo (FSE) T_1_-weighted imaging, FSE T_2_-weighted imaging, and short T_1_ inversion recovery (STIR) imaging. Subsequently, three distinct DW sequences using b = 0 and b = 600, 800 or 1000 s/mm^2^ were performed by using single-shot echo-planar imaging (SSEPI) and frequency selection fat-suppression with the same following parameters: TR/TE = 5000/72.1 ms, matrices = 128 × 128, number of excitations = 4, section thickness/interslice gap = 5/0 mm, and field of view = 320 mm. The DW series were acquired in the transverse plane and covered both breasts. Diffusion gradients were encoded in 3 orthogonal axes (x, y, and z). Acquisition time was 1 minute 20 seconds with 20-24 slices. Finally, a T_1_-weighted 3D fast spoiled gradient-recalled echo sequence with parallel imaging (VIBRANT) sequence was used for dynamic contrast-enhanced imaging. Precontrast T_1_-weighted images were subtracted from the postcontrast T_1_-weighted dynamic images on a pixel-by-pixel basis.

### Image analysis and data acquisition

All images were transferred to a workstation (Advantage Windows, version 4.2; GE Healthcare) for analysis, and the DW images were postprocessed with commercial software (FuncTool, GE Healthcare) to obtain ADC maps. Each patient had 3 ADC maps created using two b values, 0 and either 600, 800, or 1000 s/mm^2^. Two radiologists (both with 5 years of experience in breast imaging; 11 and 6 years of experience in MRI, respectively) independently evaluated each DW image and ADC map alone. To resolve disagreement between observers, a third radiologist (with 5 years of experience in breast imaging; 20 years of experience in MRI) assessed all involved items. The majority opinion was used for analysis.

Each reviewer graded the conspicuity of lesions on a 5 point confidence scale based on the appearance and signal strength of lesions on the high b value DW image (Table 1).

**Table 1:** Conspicuity grades of breast lesions on the high b value DW imaging

**Grade**	**Description**
1 = not seen	Isointensity (symmetric signal intensity of bilateral breast)
2 = probably seen	Nonlocalized, mild to moderate signal with indistinct margin
3 = seen	Circumscribed, mild to moderate signal with definite margin
4 = readily seen	Nonlocalized, strong signal with indistinct margin
5 = well seen	Circumscribed, strong signal with definite margin

Signal intensity (SI) was recorded on the high b value DW imaging and ADC was documented. Circular regions of interest (ROIs) were drawn manually in the central region of the homogeneous lesions, or in the hypointensity region of the heterogeneous lesions on ADC maps using narrow window width. Lesion signal intensity (SI_lesion_) and ADC were then automatically calculated. Signal intensity (SI_normal_) and ADC of normal tissue were measured by placing ROIs in homogeneous breast parenchyma without enhancement in the centre of the contralateral breast, avoiding contamination by fatty tissue. Finally, ROIs were drawn manually in the anterior area outside of the breast to measure noise intensity (SI_noise_) and standard deviation (SD_noise_). The area of ROI was measured to be 10 ± 2 mm^2^.

### Pathology analysis

The surgeon and study coordinator recorded the lesion location for all patients who underwent surgery or biopsy. A pathologist then performed pathological examination blinded to imaging diagnosis. The results of anatomy and histology were correlated with the results of MRI by the study coordinator who had access to all images and clinical data of the patients, including their pathology.

### Statistical analysis

Data were analyzed using the SPSS13.0 software package (SPSS Inc, Chicago, IL, USA). Probability values of less than 0.05 were considered significant. The conspicuity grades of the lesions on 3 DW series (b = 0, 600), (b = 0, 800), (b = 0, 1000) were compared using a Friedman signed rank test. SI was analyzed by measurement of SNR and contrast-to-noise ratio (CNR). SNR was calculated by SNR = SI_lesion_/SD_noise_. CNR was calculated by CNR = (SI_lesion_-SI_normal_)/SD_noise_. Comparisons of the mean SI_lesion_, SI_normal_, SI_noise_, SNR, CNR, and ADC among different b values were performed using one-way analysis of variance (ANOVA) test. For comparison of the mean ADC between malignant and benign lesions, we used the independent samples *t*-test. Receiver operating characteristic (ROC) curve analysis was used to quantify the diagnostic accuracy of ADC measurements. Nonparametric testing was performed to compare the area under the curve (AUC).

## Results

### Lesion detection

**Figure 1:**
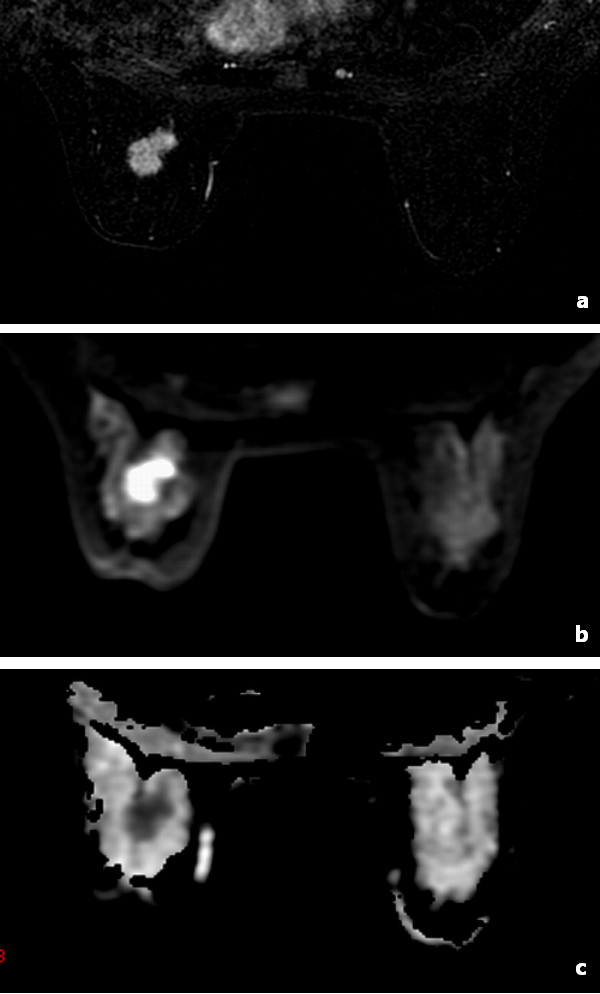
**MR images of a 49-year-old woman with invasive ductal carcinoma.** MR images of a 49-year-old woman with invasive ductal carcinoma on (**a**) early contrast-enhanced subtraction image, (**b**) gray-level DW image, and (**c**) ADC map.

Fifty-seven lesions from 52 women were analysed including 39/57 malignant (mean size, 15 mm; range, 7-45 mm) and 18/57 benign (mean size, 17 mm; range 7-50 mm) –see Table 2 for pathological subtypes. There were also 3 normal women. Contrast-enhanced imaging accurately detected 53 lesions and missed 2 hyperplasia, 1 lipoma and 1 fibroma with the sensitivity of 93.0% (53/57) and specificity of 66.7% (2/3). DW imaging accurately detected 51 lesions and missed the same four lesions, 1 other hyperplasia and 1 ductal carcinoma in situ (DCIS) with the sensitivity of 89.5% (51/57) and specificity of 100% (3/3). While the fibroma was not seen on both contrast-enhanced imaging and DW imaging, it was detected on anatomical imaging. Typical examples of the early contrast-enhanced subtraction image, DW image, and ADC map are shown in Figure1.

**Table 2:** Histological composition of malignant and benign lesions

**Malignant (*n*** **= 39)**		**Benign (*n*** **= 18)**	
IDC	33	Hyperplasia	7
Medullary carcinoma	3	Fibroadenoma	4
DCIS	3	Fibroma	1
		Lipoma	1
		Phyllodes tumors	2
		Plasmocyte mastitis	3

*IDC* invasive ductal carcinoma; *DCIS* ductal carcinoma in situ.

### Conspicuity on DW imaging

**Figure 2:**
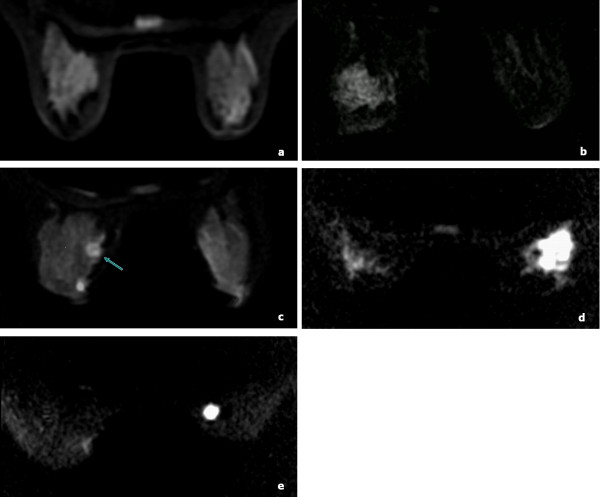
**DW images of lesions graded as 1, 2, 3, 4, and 5 point.** DW images of lesions graded as 1, 2, 3, 4, and 5 point based on the appearance and signal strength of lesions compared with surrounding breast parenchyma. (**a**) Grade 1 with symmetric signal intensity of bilateral breast. (**b**) Hyperplasia lesion in left breast scored as 2 with regional distribution, indistinct margin and moderate signal. (**c**) Fibroadenoma in left breast (arrow) scored as 3 with circumscribed appearance, definite margin and moderate signal. Another hyperintensity lesion is a cyst, which was not included in the study. (**d**) Invasive ductal carcinoma in right breast scored as 4 with segmental distribution, indistinct margin and strong signal. (**e**) Invasive ductal carcinoma in right breast scored as 5 with circumscribed appearance, definite margin and strong signal.

Most malignant lesions were circumscribed and displayed strong signals with definite margins, and benign lesions displayed mild to moderate signal with indistinct or definite margins on DW images. Mean conspicuity grades of malignant lesions were 4.44 ± 0.97 at b = 600 s/mm^2^, 4.38 ± 1.04 at b = 800 s/mm^2^ and 4.36 ± 0.96 at b = 1000 s/mm^2^. Those of benign lesions were 2.56 ± 1.38 at b = 600 s/mm^2^, 2.56 ± 1.38 at b = 800 s/mm^2^ and 2.17 ± 0.99 at b = 1000 s/mm^2^. There was a high significant difference in the conspicuity between malignant and benign lesions (*P* < 0.0001). Among 38 malignant lesions detected by DW imaging, thirty-three (86.8%) lesions showed same conspicuity grade, 3 (7.9%) darker and 2 (5.3%) brighter with increasing b values. Ten/13 (76.9%) benign lesions showed same conspicuity grade and 3/13 (23.1%) darker with increasing b values. The conspicuity grades of breast lesions were not significantly different among the three b values (*P* = 0.072). Typical DW images of five conspicuity grades are shown in Figure2.

### SI on DW imaging

SI on DW imaging depends on the magnitude of the diffusion for all lesions and normal parenchyma (Table 3). When b values increased from 600 to 1000 s/mm^2^, the average SI of malignant lesions, benign lesions, and normal parenchyma decreased from 853.2 to 473.7 (a decrease of 44.5%), 719.4 to 441.4 (38.6% reduced), and 254.2 to 150.1 (41.0% reduced), respectively. However, SI_noise_ was independent of with the change of b value. Consequently, the average SNR of malignant and benign lesions decreased significantly from 133.43 to 71.92 and 117.53 to 69.60 with higher b values, respectively (*P* < 0.001 for malignancy and *P* = 0.012 for benign lesions). Because the SI_lesion_ of malignant lesions decreased more quickly than that of benign and SI_normal_, the average CNR of malignant lesions decreased significantly with higher b values (*P* = 0.003). However, there was no significant decrease for benign lesions (*P* = 0.059).

**Table 3:** Mean SI, SNR and CNR at the three b values on DW images (Mean ± SD)

**B value (s/mm**^**2**^**)**	**Mean SI**			**SNR**		**CNR**	
	**Malignant**	**Benign**	**Normal**	**Malignant**	**Benign**	**Malignant**	**Benign**
600	853.2 ± 58.6	719.4 ± 34.4	254.2 ± 89.2	133.4 ± 20.4	117.5 ± 7.2	89.7 ± 15.9	77.3 ± 5.9
800	629.3 ± 47.5	571.6 ± 28.6	178.4 ± 60.8	97.9 ± 6.8	83.7 ± 8.9	64.1 ± 4.8	54.5 ± 8.0
1000	473.4 ± 40.3	441.4 ± 21.1	150.1 ± 45.8	69.6 ± 4.0	59.6 ± 8.9	46.0 ± 3.4	40.3 ± 7.9

*SD* standard deviation.

### ADC performance

The distribution of ADC for malignant and benign lesions calculated with different b values is shown in Table 4. Mean ADCs of malignant lesions (b = 600 s/mm^2^, 1.07 ± 0.26 × 10^-3^ mm^2^/s; b = 800 s/mm^2^, 0.96 ± 0.22 × 10^-3^ mm^2^/s; b = 1000 s/mm^2^, 0.92 ± 0.26 × 10^-3^ mm^2^/s) were significantly lower than those of benign lesions (b = 600 s/mm^2^, 1.55 ± 0.40 × 10^-3^ mm^2^/s; b = 800 s/mm^2^, 1.43 ± 0.38 × 10^-3^ mm^2^/s; b = 1000 s/mm^2^, 1.49 ± 0.38 × 10^-3^ mm^2^/s) with all *P* values <0.001. The differences in ADC calculation among the three b values were not statistically significant (*P* = 0.436). Furthermore, analysis of malignant and benign subgroups did not show significant differences in ADC calculation among the three b values, with *P* values of 0.303 and 0.840, respectively.

**Table 4:** **Mean ADCs of malignant and benign lesions at the three b values with corresponding*P*values for analyzed subgroups (×10**^**-3**^ **mm**^**2**^**/s)**

**B value (s/mm**^**2**^**)**	**Mean ADC of malignant lesions (Mean ± SD)**	**95% Confidence interval**		**Mean ADC of benign lesions (Mean ± SD)**	**95% Confidence interval**		***P*value**
		**Lower bound**	**Upper bound**		**Lower bound**	**Upper bound**	
1000	0.92 ± 0.26	0.90	1.07	1.49 ± 0.38	1.19	1.65	<0.001
800	1.96 ± 0.22	0.93	1.07	1.43 ± 0.38	1.22	1.68	<0.001
600	1.07 ± 0.26	0.98	1.15	1.55 ± 0.40	1.26	1.75	<0.001
*F* value	1.206			1.175			
*P* value	0.303			0.840			

*SD* standard deviation.

ROC curves derived from ADC differentiating malignant from benign lesions are displayed in S3. ROC analysis revealed AUC of 0.858 ± 0.075 (95% confidence interval 0.681-0.973) at b = 600 s/mm^2^, 0.870 ± 0.081 (95% confidence interval 0.672-0.988) at b = 800 s/mm^2^, and 0.866 ± 0.073 (95% confidence interval 0.692-0.976) at b = 1000 s/mm^2^. No significant difference was found among the three b values (*P* = 0.743).

**Figure 3:**
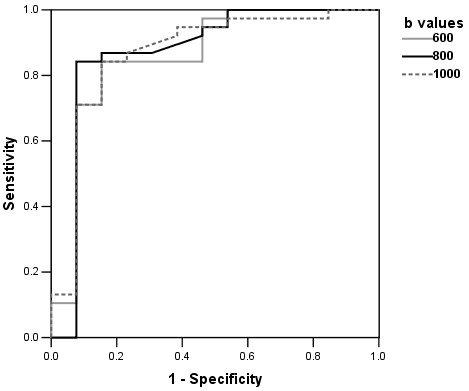
**ROC curves.** Receiver operating characteristic (ROC) curves derived from ADC in differencing malignant from benign lesions at three b values. The areas under the curves at b values of 600, 800, 1000 s/mm^2^ were 0.858, 0.870 and 0.866, respectively. There is no significant difference among three b values (*P* = 0.743).

## Discussion

DW imaging involves the random motion of water molecules, which is detected as attenuation of the measured SI [15]. The motion of water molecules is more restricted in tissues with a higher cellular density and associated with numerous intact cell membranes [5,12,24]. Therefore, highly cellular areas will appear to be higher in SI on DW images. Our study demonstrates that breast lesions displayed mild to strong SI on DW images compared to surrounding breast parenchyma, and intensity of malignant lesions was generally higher than that of benign lesions. Mean conspicuity grades of malignant lesions were 4.44 ± 0.97 at b = 600 s/mm^2^, 4.38 ± 1.04 at b = 800 s/mm^2^ and 4.36 ± 0.96 at b = 1000 s/mm^2^. Those of benign lesions were 2.56 ± 1.38 at b = 600 s/mm^2^, 2.56 ± 1.38 at b = 800 s/mm^2^ and 2.17 ± 0.99 at b = 1000 s/mm^2^. There was a highly significant difference in the conspicuity between malignant and benign lesions (*P* < 0.0001). Most malignant lesions were circumscribed and displayed strong signals with definite margins on DW images. Margin characteristics, such as the appearance of being spiculated, could not be displayed on DW images for inferior spatial resolution and partial-volume effect. Most benign lesions displayed mild to moderate signal with indistinct or definite margins on DW images. However, DW imaging cannot detect all lesions detected by other conventional MRI. This has been described previously, with 6–37.5% of malignant breast lesions reportedly not visible on DW imaging [25-27]. One DCIS and 1 hyperplastic lesion presented as non-mass enhancement on contrast-enhanced imaging but were invisible on DW imaging in the study. One fibroma displayed significant hypointensity on both T_1_WI and T_2_WI, but it was not detected on DW imaging because the signal intensity was similar to background.

DW imaging also allows quantitative calculation of ADC for each pixel of the image and is displayed as a parametric map. ADC is measured by acquiring the MR signal at least twice, typically with (S_b_) and without (S_0_) diffusion weighting by the following formula: ADC = [In (S_0_/S_b_)]/b, where S_b_ and S_0_ are the signal intensities on the DW imaging and the reference imaging without diffusion weighting, respectively [28]. Areas of restricted diffusion in highly cellular areas show low ADC compared with less cellular areas that return higher ADC values. Malignant tumours are frequently more cellular than benign lesions from which they originate and, thus, appear to be of relatively low ADC levels on ADC maps. Furthermore, ADC of invasive tumours appears to be lower than that of carcinoma in situ [5-7,9,29-31]. High-grade lesions with the highest cell proliferation rate would have the lowest ADC, which is still controversial [12,31-33]. Our results are in good agreement with previously published results on breast ADC calculation. The mean ADCs of breast cancers were significantly lower than those of benign lesions with all *P* values <0.001 at b values of 600, 800 and 1000 s/mm^2^. However, for some malignant and benign lesions, there is overlap between ADC values. This is based both on our study and the studies of others. Based on pathological analysis, 1 invasive ductal carcinoma and 1 medullary carcinoma showed ADCs that were within the confidence interval for benign lesions because of lower cellular density. One plasma cell mastitis showed a very small ADC value, possibly because of an increase in macromolecule protein and phlogocyte infiltration. Therefore, detection and characterization of breast lesions based solely on DW imaging is not sufficiently accurate, and DW imaging cannot solely substitute for other MR methods. DW imaging can be a potential adjunct to conventional MRI in the differentiation between malignant and benign lesions for fast imaging without the use of a contrast agent.

The diffusion gradient factor, also known as the “b value”, is an important parameter of DW imaging and determined by the following formula: b = γ^2^ G^2^ δ^2^(Δ – δ/3), where γ is the magnetogyric ratio, G is the gradient intensity, δ is the duration of the applied gradient, and Δ is the time interval between the paired gradients. A greater b value indicates a more severe phase dispersion of water molecules and a more reduced signal under the effect of gradient pulse on DW imaging [15]. Our study demonstrates that the average SI_lesion_ and SI_normal_ decreased with increasing b values, and SI was more severely reduced in malignant than in benign lesions and normal parenchyma. Although SNR and CNR of lesions decreased significantly, the degree was not great enough to be visually identified on DW imaging with b value increasing from 600 to 1000 s/mm^2^. Most malignant (33/38) and benign (10/13) lesions showed the same conspicuity grades on the three DW imaging. All of lesions detected on the DW imaging at b = 600 s/mm^2^ remained at significant level on the DW imaging at the higher b values although the SI of some lesions decreased with increasing b values. The conspicuity grades of breast lesions were not significantly different among the three b values (*P* = 0.072). Bogner et al. [18] also compared CNR of breast lesions at different b values on DW imaging. They have found that mean CNR for malignant and benign tumours rose with increasing b values from 0 to 850 s/mm^2^, but decreased with even higher b values from 850 to 1200 s/mm^2^. The result of our study was not consistent with that of theirs. There are two considerable differences in materials and methods compared between our study and their study. First, we performed DW imaging at 1.5 T and they performed at 3.0 T. Second, CNR were calculated differently. We directly measured SD_noise_ in the anterior areas outside of breast [34]. They calculated the standard deviation of intensities σ_*lesion*_ and σ_*tissue*_ in both volumes as SD_noise_.

We also found that there were no significant differences for ADC calculation among the three b values (*P* = 0.436). The results are consistent with previously published data [35]. Conversely, Peters et al. [36] have found that ADC of breast lesions varied substantially with the choice of different b values. However, they used quite different b values at 0, 150, 499, and 1500 s/mm^2^. ROC analysis revealed AUC of 0.858 ± 0.075 at b = 600 s/mm^2^, 0.870 ± 0.081 at b = 800 s/mm^2^ and 0.866 ± 0.073 at b = 1000 s/mm^2^ for the differentiation between malignant and benign lesions. No statistical significances were seen among the three b values (*P* = 0.743). The result is in good with previously published data involving the degree of b values ranged from 150 to 1500 s/mm^2^[35,36].

It is first to prospectively evaluate the influence of b values on both the display and ADC measurement of breast lesions on DW imaging at 1.5 T. So far, only one similar study performed using 3.0 T [18]. Our results suggest that there were significant differences in morphologic pattern and signal strength between malignant and benign lesions (*P* < 0.0001). Most malignant lesions were circumscribed and displayed strong signals with definite margins on DW images. Most benign lesions displayed mild to moderate signal with indistinct or definite margins on DW images. Other studies also demonstrated that strong signal on DW images could be help to diagnose malignant lesions, which is especially meaningful for patients who can’t accept contrast agent [37,38]. The ductus or branch distribution and the sign of ring-shape were only seen on malignant lesions in our initial study. Many articles have investigated the performance of ADC in discriminating breast lesions. However, the pooled ADC of malignant and benign lesions and their diagnostic performance vary with pathophysiologic characteristics, MRI techniques, and diagnostic criteria for malignancy in the studies. We think that the appearance pattern on DW imaging has potential value for differentiating malignant and benign breast lesions. This hypothesis should be verified by more clinical investigations.

Our study has some limitations. First, ROIs to measure SI and ADC were set by the operators, which is subjective and that little is known about the reproducibility of measurements. We found that some breast lesions were heterogeneous on DW images and ADC maps. Most tumours, especially malignant, have heterogeneous microenvironments. By drawing an ROI around a tumour, the summary statistical value does not adequately reflect lesion characteristics [15]. In order to reduce the measurement biases and avoid contamination of the data by adjacent structures, we used a fixed small size of ROI. We selected the hypointensity region of the heterogeneous lesions on ADC maps which was the highest cellular density area on histopathological specimens as ROI to avoid omitting a malignant component of any given lesion.

In addition, we combined the minimal b value of 0 mm^2^/s and the maximum b values ranging from 600 to 1000 mm^2^/s to calculate ADC. According to previously published articles, the use of multiple b values did not increase the precision of ADC measurements. Instead, it increased vulnerability of patient movement due to longer acquisition times [35]. A study performed with a 3.0 T MR imager suggested that ADC determination and DW imaging quality was optimum with a combined b value of 50 and 850 s/mm^2^[18]. Therefore, further investigation is needed to estimate whether another minimum b value, other than 0 mm^2^/s, can reduce the perfusion effect and T_2_-shine through effect on DW imaging.

## Conclusions

DW imaging is a potential adjunct to conventional breast MRI in differentiating between malignant and benign lesions. The performance of DW imaging at 1.5 T is not significantly influenced by varying the maximum b value from 600 to 1000 s/mm^2^. Further studies with larger populations are needed to confirm the use of DW imaging in the evaluation of breast lesions.

## Abbreviations

DW, Diffusion-weighted; ADC, Apparent Diffusion Coefficient; SNR, Signal-to-noise Ratio; CNR, Contrast-to-noise Ratio; ROC, Receiver Operating Characteristic; MRI, Magnetic Resonance Imaging; bmax, Maximum b Value; BI-RADS, Breast Imaging Reporting and Data System; FSE, Fast Spin-echo; STIR, Short T1 Inversion Recovery; SSEPI, Single-shot Echo-planar Imaging; SI, Signal Intensity; ROI, Regions of Interest; SIlesion, Lesion Signal Intensity; SInormal, Normal Tissue Signal Intensity; SInoise, Noise Intensity; SDnoise, Noise Standard Deviation; ANOVA, One-way Analysis of Variance; AUC, Area Under the Curve; DCIS, Ductal Carcinoma in Situ.

## Competing interests

The authors declare that they have no competing interests.

## Authors' contributions

Chen X conceived of the study concept and participated in its design, quality assessment, statistical analysis, manuscript drafting and editing and approval for important intellectual concepts. He XJ participated in its design, quality assessment, manuscript editing and approval for important intellectual concepts. Jin R carried out the MRI imaging data acquisition, participated in the literature research and helped to draft the manuscript. G YM and Zhao X participated in MRI imaging data acquisition and data analysis. Kang HF participated in its design and the clinical study. Mo LP participated in the design of the study and the pathological analysis. Wu Q participated in the design of the study and performed the statistical analysis. All authors read and approved the final manuscript.

## References

[B1] Kuhl CK Current status of breast MR imaging II. Clinical applications. Radiology 2007 244 672 691 10.1148/radiol.2443051661 17709824

[B2] Peters NH Borel Rinkes IH, Zuithoff NP, Mali WP, Moons KG, Peeters PH: Meta-analysis of MR imaging in the diagnosis of breast lesions Radiology 2008 246 116 124 18024435 10.1148/radiol.2461061298

[B3] Warner E Messersmith H Causer P Eisen A Shumak R Plewes D Systematic review: using magnetic resonance imaging to screen women at high risk for breast cancer Ann Intern Med 2008 148 671 679 18458280 10.7326/0003-4819-148-9-200805060-00007

[B4] Kuhl CK Concepts for differential diagnosis in breast MR imaging Magn Reson Imaging Clin N Am 2006 2006 14 305 328 17098173 10.1016/j.mric.2006.07.002

[B5] Guo Y Cai YQ Cai ZL Gao YG An NY Ma L Mahankali S Gao JH Differentiation of clinically benign and malignant breast lesions using diffusion-weighted imaging J Magn Reson Imaging 2002 16 172 178 12203765 10.1002/jmri.10140

[B6] Sinha S Lucas-Quesada FA Sinha U DeBruhl N Bassett LW In vivo diffusion-weighted MRI of the breast: potential for lesion characterization J Magn Reson Imaging 2002 15 693 704 12112520 10.1002/jmri.10116

[B7] Rubesova E Grell AS De Maertelaer V Metens T Chao SL Lemort M Quantitative diffusion imaging in breast cancer: a clinical prospective study J Magn Reson Imaging 2006 24 319 324 16786565 10.1002/jmri.20643

[B8] Woodhams R Matsunaga K Kan S Hata H Ozaki M Iwabuchi K Kuranami M Watanabe M Hayakawa K ADC mapping of benign and malignant breast tumors Magn Reson Med Sci 2005 4 35 42 16127252 10.2463/mrms.4.35

[B9] Marini C Iacconi C Giannelli M Cilotti A Moretti M Bartolozzi C Quantitative diffusion-weighted MR imaging in the differential diagnosis of breast lesion Eur Radiol 2007 17 2646 2655 17356840 10.1007/s00330-007-0621-2

[B10] Kuroki Y Nasu K Kuroki S Murakami K Hayashi T Sekiguchi R Nawano S Diffusion-weighted imaging of breast cancer with the sensitivity encoding technique: analysis of the apparent diffusion coefficient value Magn Reson Med Sci 2004 3 79 85 16093623 10.2463/mrms.3.79

[B11] Park MJ Cha ES Kang BJ Ihn YK Baik J The role of diffusion-weighted imaging and the apparent diffusion coefficient (ADC) values for breast tumors Korean J Radiol 2007 8 390 396 17923781 10.3348/kjr.2007.8.5.390 PMC2626812

[B12] Yoshikawa MI Ohsumi S Sugata S Kataoka M Takashima S Mochizuki T Ikura H Imai Y Relation between cancer cellularity and apparent diffusion coefficient values using diffusion-weighted magnetic resonance imaging in breast cancer Radiat Med 2008 26 222 226 18509722 10.1007/s11604-007-0218-3

[B13] Chen X Li WL Zhang YL Wu Q Guo YM Bai ZL Meta-analysis of quantitative diffusion-weighted MR imaging in the differential diagnosis of breast lesions BMC Cancer 2010 10 693 21189150 10.1186/1471-2407-10-693 PMC3024311

[B14] Tsushima Y Takahashi-Taketormi A Endo K resonance (MR) differential diagnosis of breast tumors using apparent diffusion coefficient (ADC) on 1.5-T. J Magn Reson Imaging 2009 30 249 255 19629992 10.1002/jmri.21854

[B15] Koh DM Collins DJ Diffusion-weighted MRI in the body: application and challenges in oncology AJR Am J Roentgenol 2007 188 1622 1635 17515386 10.2214/AJR.06.1403

[B16] Chiara L Diffusion and perfusion of the breast Eur J Radio 2010 76 386 390 10.1016/j.ejrad.2010.03.009 20413239

[B17] Ogura A Hayakawa K Miyati T Maeda F Imaging parameter effects in apparent diffusion coeffient determination of magnetic resonance imaging Eur J Radio 2011 77 185 188 10.1016/j.ejrad.2009.06.031 19646836

[B18] Bogner W Gruber S Pinker K Grabner G Stadlbauer A Weber M Moser E Helbich TH Trattnig S:. Diffusion-weighted MR for differentiation of breast lesions at 3.0 T: how does selection of diffusion protocols affect diagnosis? Radiology 2009 253 341 351 19703869 10.1148/radiol.2532081718

[B19] Delano MC Cooper TG Siebert JE Potchen MJ Kuppusamy K High-b-value diffusion-weighted MR imaging of adult brain: image contrast and apparent diffusion coefficient map features AJNR Am J Neuroradiol 2000 21 1830 1836 11110534 PMC7974278

[B20] Burdette JH Durden DD Elster AD Yen YF High b-value diffusion-weighted MRI of normal brain J Comput Assist Tomogr 2001 25 515 519 11473179 10.1097/00004728-200107000-00002

[B21] Kim HJ Choi CG Lee DH Lee JH Kim SJ Suh DC High-b-value diffusion-weighted MR imaging of hyperacute ischemic stroke at 1.5T AJNR Am J Neuroradiol 2005 26 208 215 15709115 PMC7974086

[B22] Burdette JH Elster AD Diffusion-weighted imaging of cerebral infarctions: are higher B values better? J Comput Assist Tomogr 2002 26 622 627 12218831 10.1097/00004728-200207000-00026

[B23] Matsuoka A Minato M Harada M Kubo H Bandou Y Tangoku A Nakano K Nishitani H Comparison of 3.0- and 1.5-tisla diffusion-weighted imaging in the visibility of breast cancer. Radiat Med 2008 26 15 20 18236129 10.1007/s11604-007-0187-6

[B24] Hatakenaka M Soeda H Yabuuchi H Matsuo Y Kamitani T Oda Y Tsuneyoshi M Honda H Apparent diffusion coefficients of breast tumors: clinical application Magn Reson Med Sci 2008 7 23 29 18460845 10.2463/mrms.7.23

[B25] Kinoshita T Yashiro N Ihara N Funatu H Fukuma E Narita M Diffusion-weighted half-Fourier single-shot turbo spin echo imaging in breast tumors: differentiation of invasive ductal carcinoma from fibroadenoma J Comput Assist Tomogr 2002 26 1042 1046 12488758 10.1097/00004728-200211000-00033

[B26] Yoshikawa MI Ohsumi S Sugata S Kataoka M Takashima S Kikuchi K Mochizuki T Comparison of breast cancer detection by diffusion-weighted magnetic resonance imaging and mammography Radiat Med 2007 25 218 223 17581710 10.1007/s11604-007-0128-4

[B27] Baltzer PA Renz DM Herrmann KH Dietzel M Krumbein I Gajda M Camara O Reichenbach JR Kaiser WA Diffusion-weighted imaging (DWI) in MR mammography (MRM): clinical comparison of echo planar imaging (EPI) and half-Fourier single-shot turbo spin echo (HASTE) diffusion techniques Eur Radiol 2009 19 1612 1620 19288109 10.1007/s00330-009-1326-5

[B28] Herneth AM Guccione S Bednarski M Apparent diffusion coefficient: a quantitative parameter for in vivo tumor characterization Eur J Radiol 2003 45 208 213 12595105 10.1016/s0720-048x(02)00310-8

[B29] Woodhams R Matsunaga K Iwabuchi K Kan S Hata H Kuranami M Watanabe M Hayakawa K Diffusion-weighted imaging of malignant breast tumors: the usefulness of apparent diffusion coefficient (ADC) value and ADC map for the detection of malignant breast tumors and evaluation of cancer extension J Comput Assist Tomogr 2005 29 644 649 16163035 10.1097/01.rct.0000171913.74086.1b

[B30] Partridge SC Mullins CD Kurland BF Allain MD DeMarini WB Eby PR Lehman CD Apparent diffusion coefficient values for discriminating benign and malignant breast MRI lesions: effects of lesion type and size AJR Am J Roentgenol 2010 194 1664 1673 20489111 10.2214/AJR.09.3534

[B31] Costantini M Belli P Rinaldi P Bufi E Giardina G Franceschini G Petrone G Bonomo L Diffusion-weighted imaging in breast cancer: relationship between apparent diffusion coefficient and tumor aggressiveness Clin Radiol 2010 65 1005 1012 21070905 10.1016/j.crad.2010.07.008

[B32] Iima M Le Bihan D Okumura R Okada T Fujimoto K Kanao S Tanaka S Fujimoto M Sakashita H Togashi K Apparent diffusion coefficient as an MR imaging biomarker of low-risk ductal carcinoma in situ: a pilot study Radiology 2011 260 364 372 21633054 10.1148/radiol.11101892

[B33] Choi SY Chang YW Park HJ Kim HJ Hong SS Seo DY Correlation of diffusion-weighted imaging apparent diffusion coefficient with prognostic factors of breast cancer Br J Radiolin press 10.1259/bjr/79381464 PMC3587081 22128125

[B34] Yang ZH Yang ZH, Feng F, Wang XY A guide to technique of magnetic resonance imaging: criterion of examination, clinical strategy and application of new techniques Quality control and artifacts processing 2010 2 People’s military medical press, Beijing 424 425

[B35] Pereira FPA Martins G Figueiredo E Domingues MN Domingues RC da Fonseca LM Gasparetto EL Assessment of breast lesions with diffusion-weighted MRI: comparing the use of different b values AJR Am J Roentgenol 2009 193 1030 1035 19770326 10.2214/AJR.09.2522

[B36] Peters NH Vinvken KL van den Bosch MA Luijten PR Mali WP Bartels LW Quantitative diffusion weighted imaging for differentiation of benign and malignant breast lesions: the influence of the choice of b-values J Magn Reson Imaging 2010 31 1100 1105 20432344 10.1002/jmri.22152

[B37] Rahbar H Partridge SC Eby PR DeMartini WB Gutierrez RL Peacock S Lehman CD Characterization of ductal carcinoma in situ on diffusion weighted breast MRI Eur Radiol 2011 21 2011 2019 21562806 10.1007/s00330-011-2140-4

[B38] Inoue K Kozawa E Mizukoshi W Tanaka J Saeki T Kimura F Usefulness of diffusion-weighted imaging of breast tumours: quantitative and visual assessment Jpn J Radiol 2011 29 429 436 21786099 10.1007/s11604-011-0575-9

